# Supplementary material for: 1-Tetradecanol, Diethyl Phthalate and Tween 80 Assist in the Formation of Thermo-Responsive Azoxystrobin Nanoparticles
Source: Molecules. 2022 Nov 17;27(22):7959. doi: 10.3390/molecules27227959 (PMC9694888; doi:10.3390/molecules27227959)
Supplement: Supplementary file 1 [file molecules-27-07959-s001.zip › molecules-1996273-supplementary.pdf]

Table S1. Xanthan gum concentrations and stirring speeds set for thermal-response AZO-loaded NPs preparation

Sample Names	Xanthan gum (‰, wt/v)	Stirring speeds (rpm, ×1000)	Xanthan gum water solution（ml）
T2t-01	0	2.0	79.0
T2t-02	0	6.0	79.0
T2t-03	0	10.0	79.0
T3t-01	0	2.0	81.5
T3t-02	0	6.0	81.5
T3t-03	0	10.0	81.5
T4t-01	0	2.0	82.0
T4t-02	0	6.0	82.0
T4t-03	0	10.0	82.0
T2t-11	0.1	2.0	79.0
T2t-12	0.1	6.0	79.0
T2t-13	0.1	10.0	79.0
T2t-21	0.5	2.0	79.0
T2t-22	0.5	6.0	79.0
T2t-23	0.5	10.0	79.0
T2t-31	1.0	2.0	79.0
T2t-32	1.0	6.0	79.0
T2t-33	1.0	10.0	79.0
T3t-11	0.1	2.0	81.5
T3t-12	0.1	6.0	81.5
T3t-13	0.1	10.0	81.5
T3t-21	0.5	2.0	81.5
T3t-22	0.5	6.0	81.5
T3t-23	0.5	10.0	81.5
T3t-31	1.0	2.0	81.5
T3t-32	1.0	6.0	81.5
T3t-33	1.0	10.0	81.5
T4t-11	0.1	2.0	82.0
T4t-12	0.1	6.0	82.0
T4t-13	0.1	10.0	82.0
T4t-21	0.5	2.0	82.0
T4t-22	0.5	6.0	82.0
T4t-23	0.5	10.0	82.0
T4t-31	1.0	2.0	82.0
T4t-32	1.0	6.0	82.0
T4t-33	1.0	10.0	82.0
CK0	0.1	10.0	82.0
CK1	0.1	10.0	82.0

Table S2. Test for suppressing efficacy of T3t-c12 and T3t-13 NPs on germination of A1513 spores.

Samples	T3t-c12, 10% AZO (μl)	T3t-13, 5% AZO (μl)	Final AZO amount (μg/plate)
AT3-c100	4.0	0	100.0
AT3-c80	3.2	0	80.0
AT3-c60	2.4	0	60.0
AT3-c40	1.6	0	40.0
AT3-c20	0.8	0	20.0
AT3-100	0	8.0	100.0
AT3-80	0	6.4	80.0
AT3-60	0	4.8	60.0
AT3-40	0	3.2	40.0
AT3-20	0	1.6	20.0
CK3	0	0	0

Method S1. Culture of PNgz07 and A1513 strains.

PNgz07 and A1513 strains were cultured on Tab-5 medium and PDA medium, respectively. The Tab-5 medium was prepared as following: 30.0 g potato powder, 5.0 g calf extract, 1.5 g $MgSO_4$, 3.0g K_2HPO_4, 20.0 g glucose, and 20.0 mg VB1, 6.0 g yeast extract, 2.0 g peptone, 14.0 g agar powder, and 25.0 g nutrient soil were dissolved or suspended in 1000 ml *d*H_2O and sterilized in an autoclave at 121°C for 30 min. PDA medium was prepared according to the guideline of commercial product.

Both PNgz07 and A1513 strains were inoculated on agar plates and cultured at 30 ^oC in dark except special description in main body of paper.

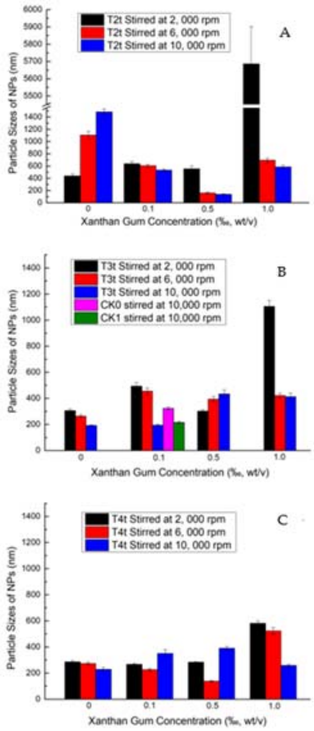

Figure S1. Particle sizes of AZO NPs prepared with nonionic emulsifier - tween 80. A: particle sizes of T2t NPs, B particle sizes of T3t, CK0 and CK1 NPs, C: particle sizes of T4t NPs.

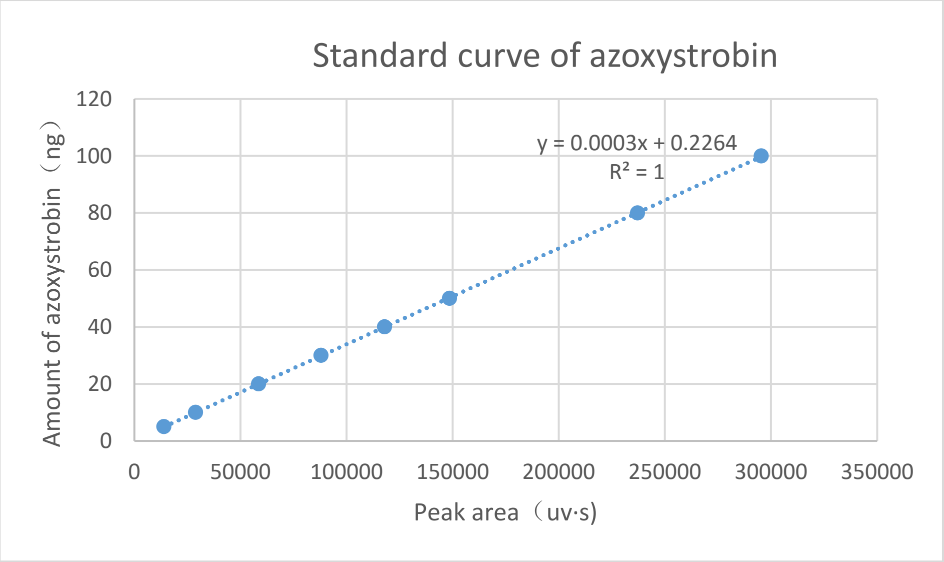

Figure S2: Standard curve of AZO under determination conditions on HPLC.
